# Evaluating Clinical Outcomes for Determining the Optimal Delay to Skin Incision under WALANT: A Prospective Series of 34 Patients from a Low-Resource Tertiary Setting

**DOI:** 10.1155/2020/9351354

**Published:** 2020-08-15

**Authors:** Alvin Hernandez, Mamer Rosario, Romina Mendoza-Torres, Carl Ryan Marino Taguba, Abigail Garcia, Geoffrey Battad

**Affiliations:** Department of Orthopaedics, East Avenue Medical Center, East Avenue, Diliman, Quezon City 1101, Metro Manila, Philippines

## Abstract

**Background:**

Additional studies on clinical outcomes to determine the optimal time delay from injection of local anesthesia to skin incision for WALANT surgeries are needed. The authors aimed to propose the optimal time delay from local injection to skin incision for WALANT surgeries of the hand and wrist by analyzing intraoperative blood loss, postoperative pain scores, and complication rates.

**Methods:**

Thirty-four patients were consecutively recruited and allocated by either 7-min or 30-min delay for skin incision from local injection of epinephrine with lidocaine. Intraoperative bleeding and postoperative pain scores were analyzed between both groups by Mann–Whitney *U*-test, while complication rates were compared using Fisher's exact test.

**Results:**

The present study did not find significant differences in mean intraoperative blood loss (8 ± 5.8 mL vs. 5 ± 2.2 mL, *p*=0.074), complication rates (18% vs. 0%, *p*=0.227), and mean pain scores (1.2 ± 0.5 vs. 1.4 ± 0.5, *p*=0.307) between the 7-min and 30-min groups.

**Conclusion:**

The authors conclude that a waiting time of 7 min from the injection of local anesthesia is sufficient to achieve comparable clinical outcomes for minor hand and wrist surgeries under WALANT.

## 1. Introduction

Hand surgeries traditionally rely on tourniquet to minimize intraoperative bleeding. However, upper arm tourniquet may cause pain and discomfort among wide-awake patients without general anesthesia or brachial plexus block [1, 2]. Minor procedures of the hand and wrist can be performed with local anesthesia without sedation on an outpatient basis. Use of upper arm tourniquet, however, can limit the duration of procedure due to excessive pain caused on some patients [2–4].The wide-awake local anesthesia no tourniquet (WALANT) technique [5], in which lidocaine and epinephrine are injected for local anesthesia and vasoconstriction, respectively, has been increasingly used for hand surgeries [1, 6, 7].The WALANT technique enables the surgery to be performed with the patient fully awake and without upper arm tourniquet, which allows intraoperative assessment of function during the procedure [1, 5].

Bleeding can be minimized with the WALANT technique by waiting for the optimal epinephrine effect after local anesthetic injection. Although vasoconstriction by epinephrine is traditionally believed to be optimal at approximately 7 to 10 minutes (min) from the time of injection [8], McKee et al. [8], in a prospective randomized trial, obtained the lowest cutaneous hemoglobin levels at roughly 26 min following injection of epinephrine. In their follow-up prospective comparative series [9] on carpal tunnel surgeries performed by WALANT, waiting roughly 30 min after injection of epinephrine resulted near three-fold reduction in mean quantity of intraoperative bleeding.

However, to the best of our knowledge, no additional studies on clinical outcomes to determine the optimal time delay to skin incision for WALANT surgeries of the hand or wrist have been published. In this regard, our study aimed to propose the optimal time delay from the injection to skin incision for most WALANT surgeries of the hand or wrist by analyzing not only intraoperative blood loss but also postoperative pain scores and complication rates, in a larger single-institutional cohort of patients.

## 2. Patients and Methods

In this prospective comparative study, healthy patients undergoing minor hand or wrist surgeries by WALANT on an outpatient basis at the East Avenue Medical Center, a public tertiary hospital in the Philippines, waited either 7 min or 30 min from the time of injection to the time of skin incision. Institutional review board approval was obtained. Patients undergoing repeat surgery, on anticoagulation medications, and with bleeding disorders were excluded. Thirty-four patients were consecutively recruited from January 1 to December 31, 2019, with our sample size based on the calculation performed by McKee et al. [8] for their study. The patients were not formally randomized but allocated prior to arriving at the operating room, based on surgeon scheduling factors. For 17 patients, we waited exactly 7 min following injection of local anesthesia before cutting, with each interval timed using a stopwatch, and then recorded. While for the other 17 patients, we waited exactly 30 min prior to skin incision.

For local anesthesia, we used up to 10 mL [10] of 1% lidocaine with 1 : 100,000 concentration of epinephrine (OCTOCAINE^**®**^; Novocol Pharma, Inc., Cambridge, Canada) introduced in a subcutaneous plane along the length of the planned incision. The same injector (AH) provided the anesthesia for all patients. All procedures were performed without the aid of surgical loupes. Intraoperative blood loss was estimated for each procedure using the method developed by Algadiem et al. [11]. Pain was assessed using the Verbal Rating Scale (VRS) [12], recorded from each patient at 1 hour after the procedure. Perioperative complications were documented and recorded. Median follow-up after surgery is 14 days (range 14 to 28 days).

Both the mean intraoperative blood loss in mL and mean VRS scores for both groups were analyzed using the Mann–Whitney *U*-test, while complication rates were compared using Fisher's Exact test. All statistical analyses were performed using SPSS version 21.0 (IBM Corp., Armonk, NY), with a *p* value of less than 0.05 considered statistically significant.

## 3. Results

We categorized patient-, disease-, and treatment-related variables according to the time delay from the injection of local anesthesia to skin incision (Table 1). The 7-min group and the 30-min group had a mean age of 48 years (range, 31 to 75 years) and 53 years (range, 39 to 75 years), respectively, and a male-to-female ratio of 1 : 1.12 for both groups. The mean time intervals of surgery from skin incision to closure, for the 7-min and 30-min groups, are 7.5 min (range, 6 to 12 min) and 6.8 min (range, 5 to 11 min), respectively, while the mean length of skin incision is 1.7 cm for both groups. Mean VRS scores at 1 hour postsurgery for the 7-min and 30-min groups are 0.6 (range, 0 to 1) and 0.5 (range, 0 to 1), respectively, while mean intraoperative bleeding for the 7-min and 30-min groups are 8 mL (range, 3 to 27 mL) and 5 mL (range, 3 to 12 mL), respectively. Three complications, one intraoperatively and two postoperatively, were documented in the 7-min group, while there was none in the 30-min group. Specifically, iatrogenic digital vessel injury during surgery, wound dehiscence, and development of neuroma occurred in three cases of trigger finger release (Table 2).

Our study did not find statistically significant differences in mean intraoperative blood loss (8 ± 5.8 mL vs. 5 ± 2.2 mL, *p*=0.074), complication rates (18% vs. 0%, *p*=0.227), and mean VRS scores (1.2 ± 0.5 vs. 1.4 ± 0.5, *p*=0.307) between the 7-min and the 30-min groups (Table 3).

## 4. Discussion

When performing WALANT surgeries, waiting for the optimal epinephrine effect after local anesthetic injection shall minimize bleeding. Despite data by some authors [6, 7] showing significant reduction in intraoperative blood loss by waiting approximately 30 min from the time of epinephrine injection before incising skin, literature on clinical outcomes determining the optimal time delay to skin incision is scarce. Our study sought to propose the optimal time delay from injection to skin incision for most WALANT surgeries of the hand or wrist by analyzing clinical outcomes, particularly pain scores and complication rates, in addition to intraoperative blood loss.

The study has several limitations. First, our use of McKee et al.'s method [9] for measuring blood loss, although reliable, must be less accurate than that performed by other authors [9].However, we estimated the total intraoperative blood loss for each procedure, from skin incision to closure, to have better measure of intraoperative bleeding. Second, we included all hand and wrist surgeries in the analysis, instead of controlling confounders by limiting to a single hand procedure, like carpal tunnel release [8, 9]. Nonetheless, we upheld homogeneity of both groups, having found no significant differences in mean incision length (1.7 ± 0.5 cm vs. 1.7 ± 0.4 cm, *p*=0.807), mean interval duration from skin incision to closure (7.5 ± 1.7 min vs. 6.8 ± 1.3 min, *p*=0.242), and mean preoperative VRS scores (1.2 ± 0.7 vs. 1.4 ± 0.7, *p*=0.320) between the 7-min and 30-min groups (Table 4). Third, randomization and blinding were not done. Although we have contributed additional data from a larger prospective cohort, more randomized controlled trials to fill the present lack of studies are recommended. Fourth, we did not use lidocaine for control measurements of bleeding. It has been reported by some authors [13, 14] that lidocaine has an initial vasodilatory effect in the skin. However, recent studies [15, 16] have shown no changes in perfusion following lidocaine infiltration on porcine eyelid flaps and flanks. Fifth, our study is limited by the relatively short follow-up period. Given our setting, it must be financially taxing for patients to pursue regular follow-up after surgery. In our experience, patients most commonly seek follow-up only for either the removal of wound sutures or development of a complication. A patient's nonappearance during follow-up somehow reassures there must be no postoperative complications. Lastly, multi-institutional studies involving larger cohorts must be necessary, in order to address the relatively small number of patients included in our analysis.

Having found no significant increase in blood loss with the 7 min delay from injection of local anesthesia, our findings seem in contrast with the controversial results of McKee et al. [8, 9].Although the disparity between our results and those of McKee et al.'s [9] study may be due to differences in the method of blood collection (by micropipette versus surgical gauze [11]), number of cases (15 versus 34), and length of skin incision (2.5 to 3 cm versus 1.7 cm), our findings must reflect the better estimates of total intraoperative bleeding since we opted to collect blood throughout the whole duration of each procedure (versus the 1 min interval by McKee et al. [9]). Therefore, a waiting time of 7 min seems acceptable, and sufficient, to reduce intraoperative bleeding when performing minor hand and wrist surgeries under WALANT. The epinephrine concentration used (1 : 100,000), anatomical location (hand and wrist), and patient demographics (age and sex) were similar in both studies.

Our findings are, moreover, in agreement with those by preceding authors [14–19]. Larrabee et al. [17] injected epinephrine with either lidocaine or saline into the skin of piglet trunks and found maximal vasoconstriction, measured by laser Doppler flowmetry, to occur at about 5 to 7 min in most of the cases. Similarly, O'Malley et al. [18] recorded maximal vasoconstriction following epinephrine injection in the neck of patients undergoing head and neck surgery after 3 to 4 min, while Ghali et al. [14] found maximal decrease in blood flow following injection of epinephrine to happen after 8 min in the face and 10 min in the forearm. Additionally, Hult et al. [19] estimated intraoperative bleeding in the eyelids of 16 patients undergoing upper eyelid blepharoplasty and found significant decrease in bleeding at 7 min following epinephrine injection, with no further decrease at 15 and 30 min thereafter. In a recent study by Sheikh et al. [15] on porcine flank flaps injected with epinephrine and lidocaine, low level of perfusion, measured by laser speckle contrast imaging and laser Doppler flowmetry, was already observed after approximately 2 minutes following the injection. In a similar study by the same authors [16] on porcine eyelid flaps, time to maximum hypoperfusion was found even shorter, recorded by laser speckle contrast imaging at 1.25 min following the injection.

The present study is the first to evaluate complication rates and pain scores for determining the optimal time delay from local anesthesia to skin incision when performing the WALANT technique. Despite the three complications that occurred in patients from the 7-min group, we found no significant difference in complication rates between the two groups. The digital vessel injury was adequately controlled by coagulation with direct pressure, while the wound dehiscence was formally debrided and then closed by suture repair. The neuroma was conservatively treated with 150 mg of oral pregabalin (LYRICA^**®**^; Pfizer, Inc., New York City, USA) daily for a week, which completely resolved the pain thereafter. Huang et al. [1], in a retrospective analysis of 24 distal radius fractures treated by open reduction and plating under WALANT, documented no complications following a mean follow-up period of 15 months. In contrast to Huang et al.'s study, the complication rate (9%) we found in our series must be high. We additionally analyzed a total of 19 patients who underwent minor hand procedures in our institution, but under local anesthesia with tourniquet in the same study period, and documented two (11%) complications after a median follow-up of 14 days (range, 14 to 121 days). Upon comparison with our WALANT cohort, no statistically significant difference in complication rates (9% vs. 11%, *p*=1.0) was found. Aside from having analyzed nonfracture WALANT cases, the development of complications in our series must be explicable in the setting of a public tertiary facility in a developing country [20, 21].In our retrospective cohort that underwent surgery under local anesthesia with tourniquet, superficial surgical site infection and finger stiffness developed in two cases of trigger finger release. The former was successfully treated with 2-week regimen of 500 mg oral cefuroxime (CEFUREX^**®**^; Cathay Drug Co. Inc., Makati City, PH) twice daily and the latter, with six weeks of physical therapy. It has been recognized that traditional clinical research using standard follow-up is extraordinarily difficult in the developing world, where resources are limited [22]. It is financially challenging for our patients to pursue regular follow-up after surgery, and similar to the experiences of Young et al. [23], patients most commonly seek follow-up for the removal of wound sutures or due to development of a complication and less for surveillance. Moreover, we performed all procedures without the aid of surgical loupes, which could have promoted optimal tissue handling and dissection, and thus prevented iatrogenic soft tissue injury and wound complications. Although the low postoperative pain scores we found, in addition to those found by Huang et al. in their study [1], they must lay support to the latter authors' findings of significantly lower mean postoperative pain score under WALANT, in their subsequent comparative study on 47 distal radius fractures treated by volar plating, under either WALANT or general anesthesia with arm tourniquet [7].

## 5. Conclusion

In conclusion, we showed that a waiting time of 7 min from the injection of local anesthesia is sufficient to achieve comparable clinical outcomes for minor hand and wrist surgeries under WALANT. However, findings of the present study must be considered preliminary, and randomized clinical trials for larger cohort analyses are deemed necessary.

## Figures and Tables

**Table 1:** Patient characteristics.

	Total, *N* = 34	
	7-min (*n* = 17)	30-min (*n* = 17)
Age (years) ^*∗*^	48 (31 to 75)	53 (39 to 75)
Gender ^*∗∗*^		
Men	8 (47)	8 (47)
Women	9 (53)	9 (53)
Procedure ^*∗∗*^		
Trigger finger release	13 (76)	12 (70)
Carpal tunnel release	3 (18)	3 (18)
Excision of mass	1 (6)	2 (12)
Interval from skin incision to closure (min) ^*∗*^	7.5 (6 to 12)	6.8 (5 to 11)
Length of incision (cm) ^*∗*^	1.7 (1.5 to 3)	1.7 (1.5 to 2.5)
Preoperative VRS score ^*∗*^	1.2 (0 to 2)	1.4 (0 to 2)
VRS score 1-hour postsurgery ^*∗*^	0.6 (0 to 1)	0.5 (0 to 1)
Intraoperative blood loss (ml) ^*∗*^	8 (3 to 27)	5 (3 to 12)
Perioperative complications ^*∗∗*^	3 (18)	0 (0)

^*∗*^Presented as the mean with the range in parentheses.  ^*∗∗*^Presented as the number with the percentage in parentheses.

**Table 2:** List of perioperative complications.

Case no.	Group	Age/sex	Operation	Complication	Management	Outcome
13	7-min	53/F	Trigger finger release	Wound dehiscence	Debridement and primary closure	Improved
17	7-min	51/M	Trigger finger release	Digital vessel injury	Direct pressure	Improved
20	7-min	54/F	Trigger finger release	Painful neuroma	Oral pregabalin	Improved

**Table 3:** Analyses of intraoperative bleeding, postoperative VRS scores, and complication rates.

	*N*	Mean/proportion	*p* value
Blood loss			0.074
7-min	17	8 mL	
30-min	17	5 mL	

Postoperative VRS scores			0.307
7-min	17	1.2	
30-min	17	1.4	

Complications			0.227
7-min	17	18%	
30-min	17	0%	

**Table 4:** Analyses of preoperative VRS scores, duration of procedure, and length of skin incision.

	*N*	Mean/proportion	*p* value
Preoperative VRS scores			0.320
7-min	17	1.2	
30-min	17	1.4	

Duration of procedure			0.242
7-min	17	7.5 min	
30-min	17	6.8 min	

Length of skin incision			0.807
7-min	17	1.7 cm	
30-min	17	1.7 cm	

## Data Availability

The data used to support the findings of this study are included within the supplementary information file.

## Supplementary Materials

Supplementary Materials List of Minor Hand Surgeries done at our institution from January–December 2019. Surgeries performed were labeled as either WALANT or non-WALANT cases.
